# Exosomes in Severe Asthma: Update in Their Roles and Potential in Therapy

**DOI:** 10.1155/2018/2862187

**Published:** 2018-05-08

**Authors:** Esmaeil Mortaz, Shamila D. Alipoor, Mohammad Varahram, Hamidreza Jamaati, Johan Garssen, Sharon E. Mumby, Ian M. Adcock

**Affiliations:** ^1^Department of Immunology, Faculty of Medicine, Shahid Beheshti University of Medical Sciences, Tehran, Iran; ^2^Clinical Tuberculosis and Epidemiology Research Center, National Research Institute of Tuberculosis and Lung Diseases, Shahid Beheshti University of Medical Sciences, Tehran, Iran; ^3^Institute of Medical Biotechnology, Molecular Medicine Department, National Institute of Genetic Engineering and Biotechnology (NIGEB), Tehran, Iran; ^4^Mycobacteriology Research Center, National Research Institute of Tuberculosis and Lung Diseases (NRITLD), Shahid Beheshti University of Medical Sciences, Tehran, Iran; ^5^Chronic Respiratory Diseases Research Center, National Research Institute of Tuberculosis and Lung Diseases, Shahid Beheshti University of Medical Sciences, Tehran, Iran; ^6^Division of Pharmacology, Utrecht Institute for Pharmaceutical Sciences, Faculty of Science, Utrecht University, Utrecht, Netherlands; ^7^Nutricia Research Centre for Specialized Nutrition, Utrecht, Netherlands; ^8^Cell and Molecular Biology Group, Airways Disease Section, Faculty of Medicine, National Heart and Lung Institute, Imperial College London, London, UK; ^9^Priority Research Centre for Asthma and Respiratory Disease, Hunter Medical Research Institute, University of Newcastle, Newcastle, NSW, Australia

## Abstract

Exosomes are nanosized vesicles and have recently been recognized as important players in cell-to-cell communication. Exosomes contain different mediators such as proteins, nucleic acids (DNA, mRNA, miRNAs, and other ncRNAs), and lipid mediators and can shuttle their exosomal content to both neighboring and distal cells. Exosomes are very effective in orchestrating immune responses in the airways and all cell types can contribute to the systemic exosome pool. Intracellular communication between the broad range of cell types within the lung is crucial in disease emphasizing the importance of exosomes. In asthma, exosomes affect the inflammatory microenvironment which ultimately determines the development or alleviation of the pathological symptoms. Recent studies in this area have provided insight into the underlying mechanisms of disease and led to interest in using exosomes as potential novel therapeutic agents.

## 1. Introduction

Asthma is a heterogeneous syndrome involving inflammation and obstruction of the airways that affects 300 million people worldwide [1, 2]. Limited knowledge of the disease mechanisms is the greatest obstacle to the development of novel treatments. Although two forms of asthma have been traditionally defined in the clinic (T2 and non-T2), this ignores the broad range of phenotypes that have been described and the underlying pathophysiology of these phenotypes. As a result, asthma is increasingly recognized as a syndrome rather than a single disease [3, 4]. The goal of asthma research is to link asthma classification based on phenotypes with pathophysiological mechanism and thereby define asthma endotypes which will predict drug efficacy [4]. Several asthma phenotypes have been described such as allergic bronchopulmonary mycosis and severe late-onset hypereosinophilic asthma [4, 5]; however, a small group of patients have asthma that is uncontrolled or only partially controlled despite intensive treatment [6]. This form of asthma is commonly referred to as severe asthma [7] which is often associated with serious morbidity and even mortality [6].

The emergence of biomarkers such as blood eosinophils linked with T2-asthma targeted biologic therapies opens new hopes for patients with severe asthma. However, further research is required to understand the mechanisms underlying pathophysiology of severe non-T2 asthma and to define the optimal biological treatment. In addition to this it is important to have readily accessible biomarkers that define patient subsets to ensure that the correct drug is given to the right patient at the right time. This is essential for the patients' perspective and for the healthcare provider where the current blunt measures such as blood eosinophils do not distinguish differences in underlying pathophysiological processes.

Exosomes are small vesicles (30–100 nm in diameter) that enable cell-to-cell communication by shuttling different molecules such as nucleic acids (DNA, mRNA, and micro (mi)RNAs), lipids, proteins such as heat shock 70-kDa protein (HSP)70, and specific cell surface markers reflecting the exosome cell of origin. These would include CD9, CD63, and CD81 if the exosome was endosomal in origin [8]. Exosomes can, therefore, significantly affect target cell function resulting in the development of a pathological state [9].

Exosomes have been most extensively studied in association with the pathogenesis of diverse diseases, such as cancer [10, 11] and infectious disease [12–14] as well as in asthma [15]. Exosome biology has provided us with fundamental insights into the mechanisms of cellular crosstalk in asthma and may also act as important biomarkers of the disease. In this review we summarize recent advances regarding the roles of exosomes in the pathogenesis of severe asthma and discuss their potential as biomarkers for targeted treatments.

## 2. Asthma Pathogenesis

Asthma is a complex disease whose underlying pathophysiology is not completely understood [16]. As a chronic inflammatory airway disease, asthma involves many cells from the innate and adaptive immune systems which act on airway epithelial cells to trigger bronchial hyperreactivity and airway remodeling in response to environmental stimuli such as allergens, infections, or air pollutants [3, 17]. The main features of allergic asthma are increases in the numbers and activity of airway mast cells and eosinophils which are due to the pathophysiological effects of proinflammatory cytokines such as interleukin- (IL-) 4, IL-5, and IL-13 released by activated CD4+ T-cells (Th2 cells) in response to environmental allergens [3]. In addition to lymphocytes and plasma cells, a large number of eosinophils and neutrophils are observed in the bronchial tissues and mucus of asthmatic airways [18].

**Figure 1:**
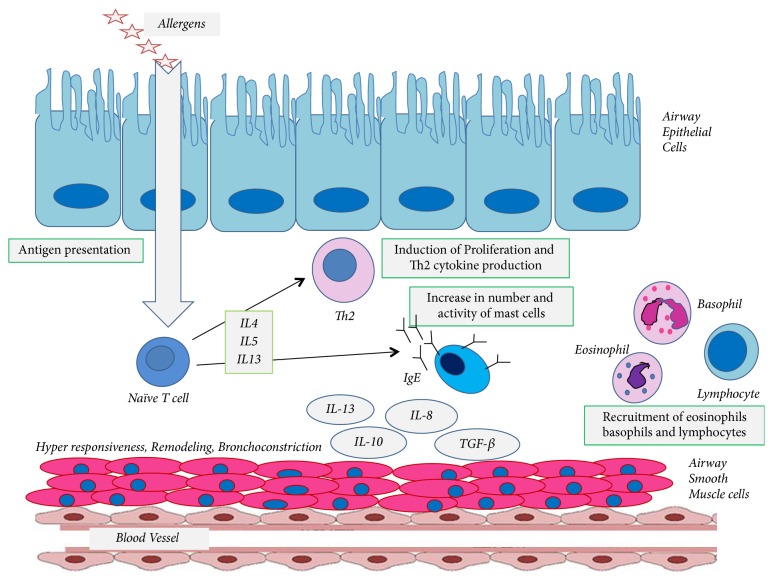
*The pathogenicity of asthma*. The entry of allergens into the airway triggers the Th2 response through the antigen presenting cells and induce the differentiation of naïve CD4+ T-cells into CD4+ Th2 cells in the presence of IL-4, IL-5, and IL-13. Activated CD4 T-lymphocytes recruit leukocytes to the airway from the bloodstream which will follow with the secretion of inflammatory mediators from eosinophils, mast cells, and lymphocytes within the airway. The expression of Th2 cytokines directs the switch from IgM to IgE antibody production. Mast cell activation and degranulation are triggered following cross-linking of the membrane bound high affinity IgE receptor on mast cells. Chronic inflammation in the asthmatic airway leads to repeated cycles of tissue injury and repair which results in structural alterations and remodeling of the airways over time.

During an asthma attack, airway provocation with allergens triggers a rapid decrease in bronchial airflow with an early immunoglobulin E- (IgE-) mediated reaction that may be followed by a late-phase IgE-mediated decrease in bronchial airflow for 4–8 hours [19]. Based on our understanding of the pathophysiology of allergic asthma, activated CD4 T-lymphocytes recruit leukocytes to the airway from the bloodstream and the presence of these stimulated leukocytes results in the secretion of inflammatory mediators from eosinophils, mast cells, and lymphocytes within the airway. The expression of Th2 cytokines from activated T-lymphocytes also directs the switch from IgM to IgE antibody production [20]. Mast cell activation and degranulation are triggered following cross-linking of the membrane bound high affinity IgE receptor (Fc*ε*RI) on mast cells which causes them to release inflammatory lipid mediators such as histamine and leukotrienes (LTs). In addition, IL-5 directs the recruitment of eosinophils from the bone marrow to the site of airway inflammation [21, 22]. Chronic inflammation in the asthmatic airway leads to repeated cycles of tissue injury and repair which results in structural alterations and remodeling of the airways over time [23, 24] (Figure 1).

## 3. Exosome Properties and Function

Exosomes are small 30–100 nm membrane-enclosed vesicles. They were discovered in 1983 and initially were described as small vesicles that bud from reticulocytes during their maturation and thought to function as the cell's “garbage bin” [8]. Further studies indicated that exosomes are released from most mammalian cells and are found in nearly all biological fluids [25]. Later studies determined the biological function of exosomes [26, 27] and highlighted their involvement in many pathological conditions such as cancer and neurodegenerative and infectious diseases as well as in immune-modulatory processes [28, 29]. The watershed moment in the study of exosomes came in 2007 with the finding that exosomes contained more than 1200 mRNAs which were translated into proteins following delivery to recipient cells [29, 30]. The crucial importance of exosomes, therefore, lies in their capacity to shuttle information between cells and influence the function of recipient cells [12]. Exosomes have also recently been implicated in cell homeostasis and the removal of unwanted molecules [31].

**Figure 2:**
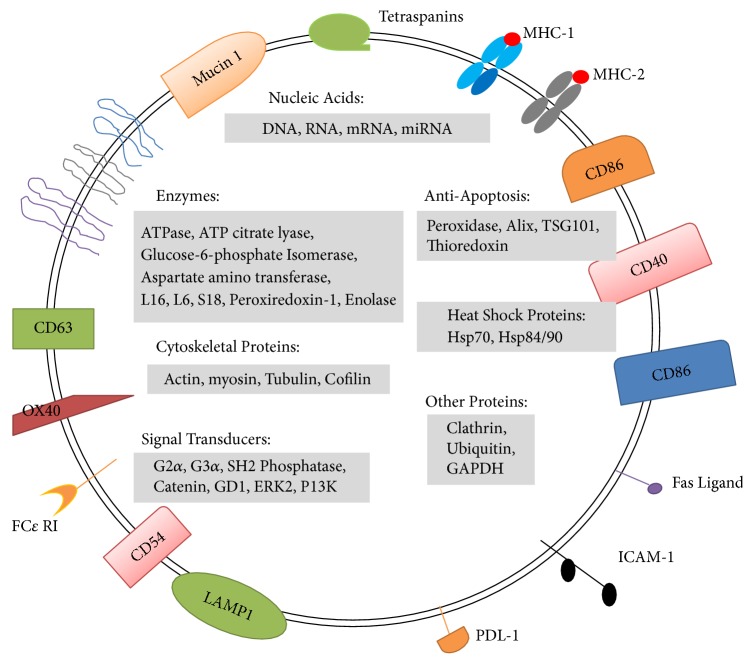
*Exosomes are small membrane-enclosed vesicles* containing mRNA and miRNA, lipids, and a vast array of different proteins depending on their cell of origin. Generally exosomes are enriched in some of generic proteins such as proteins involved in MVB formation, tetraspanins, and membrane transports as well as a number of cytosolic proteins. In addition some compounds associated with specific pathological condition have been identified in exosomes.

Exosome-derived signaling molecules include proteins, lipids, nucleic acids, and miRNAs whose packaging together gives an advantage of simultaneous delivery of multiple components to target cells [32]. An important feature of exosomes is that they are highly stable in biological fluids [33]. In addition, their content and composition resemble their cell of origin and these may change according to the physiological or pathological conditions the cell is exposed to [34]. Exosomes may contain many bioactive agents including prostaglandins and LTs, lipids, transmembrane receptors such as integrins *β*1 and *β*2, costimulatory molecules, membrane-localized classes I and II major histocompatibility complexes (MHC), signal transduction proteins, and nucleic acids (mRNA and miRNAs) [12] (Figure 2).

Extensive investigations have elucidated the role of exosomes in intercellular communication and the regulation of physiological functions and homeostasis as well as their contribution to various pathological conditions [32]. It is within this context that we review the function of exosomes in the development of asthma with particular reference to severe disease.

## 4. Exosomes in Severe Asthma

The lung is a complex organ composed of a wide range of immune and structural cells within the parenchyma and airway [35]. For optimal functioning, cell-cell communication is essential and so exosomes are expected to play crucial role in lung biology and function [36]. In relation to the pathobiology of asthma, exosomes are released from the key cells implicated in disease such as mast cells, eosinophils, dendritic cells (DCs), T-cells, and bronchial epithelial cells. These in turn can trigger the activation, or repression, of other asthma-associated cells and enhance allergic responses [37].

DC-derived exosomes have costimulatory molecules on their surfaces that can activate allergen-specific Th2 cells [33, 38]. In addition, eosinophil-derived exosomes have important roles in the modulation of asthma and their numbers are increased in asthmatic patients [39, 40]. Analysis of exosomal miRNAs in patients with severe asthma compared with healthy subjects showed an altered miRNA content. The dysregulated miRNAs were involved in pathways related to airway integrity as well as being correlated with some clinical features such as eosinophil count or FEV1 [41]. In a separate study, the differential exosomal miRNAs profile in SA patients were associated with TGF-ß signaling pathway, the ErbB signaling pathway, and focal adhesion [42].

BAL exosomes from asthmatic patients express the epithelial marker mucin 1 on their surface indicating that they are derived from bronchial epithelial cells [43]. They were able to induce the production of CXCL-8 and LT C4 in target bronchial epithelial cells [44]. Whether this is a natural autocrine effect of these exosomes or whether other cells are the physiological target cell is unknown but BAL exosomal miRNAs from asthmatics were involved in IL-13-mediated events [45]. In a feedback manner, IL-13 promotes exosome production by airway epithelial cells and these exosomes subsequently enhance the proliferation of undifferentiated lung macrophages [44]. Thus, both structural and effector cells produce exosomes that modulate the chronic inflammatory processes involved in asthma [15].

### 4.1. Exosomes from Immune Effector Cells

Inflammation is the main pathogenic driver in asthma. Exosomes can promote inflammation via regulating the function of immune cells at the level of their recruitment, activation, or differentiation. A broad range of cells in lung are involved in asthmatic inflammation including airway epithelial cells [46–48], eosinophils [39, 49], lymphocytes [32, 46, 50], macrophages [46, 51], and DCs [48].

*Eosinophils* are multifunctional granulocytes that have an important role in both allergy and asthma due to their production, storage, and release of a range of inflammatory mediators. These include chemokines, lipid mediators, and cytotoxic granule proteins such as major basic protein (MBP), eosinophil peroxidase (EPX), eosinophil cationic protein (ECP), and eosinophil-derived neurotoxin (EDN), which together result in several key features of asthma.

Eosinophils from asthma patients release a greater number of exosomes in comparison with those released from cells of healthy subjects. These exosomes contain the main eosinophilic proteins such as EPO, MBP, and ECP and may, therefore, play a similar role in driving the progression of asthma as their parent cell [39]. Eosinophil-derived exosomes isolated from asthmatics may have both autocrine and paracrine functions as they increase in the production of chemokines, reactive oxygen species (ROS), and nitric oxide (NO) from target eosinophils as well as enhancing eosinophil migration by upregulating the expression of adhesion molecules such as intercellular adhesion molecule 1 (ICAM-1) and integrin *α*2 [49] which is a critical step in asthma development [15].

*Lymphocytes* are key players in the inflammatory response in allergy and asthma. B-lymphocytes produce antigen specific immunoglobulins E (IgE) following Th2 cell activation and release of Th2 cytokines [52]. B-lymphocytes can also trigger an asthmatic response by acting as an antigen presetting cell (APC) without the involvement of IgE and T-lymphocytes [53]. In addition, B-lymphocytes are involved in the differentiation of naïve Th0-lymphocytes into Th1- or Th2-lymphocytes by releasing IFN-*γ* or IL-4, respectively [54]. Finally, IL-10-producing B-cells or Breg (B regulatory) downregulate inflammation in hyperresponsiveness airway and suppress allergic inflammation by recruitment of natural Treg (CD4+ CD25+ FoxP3+) cells to the lung [55].

B-cell-derived exosomes resemble their parent phenotype and carry MHC classes I and II and integrins *β*1 and *β*2 as well as the costimulatory molecules CD40, CD80, and CD86. As a result, they can specifically present antigenic peptides to T-cells and induce T-cell responses [56]. B-cell exosomes also contain HSP70 which is important in DC maturation [57]. B-cell-derived exosomes can also modulate the proliferation and production of Th2 cytokines from T2 cells due to the presence of exosomal antigens such as birch peptide (Bet v1) to the same degree as observed upon direct contact between B- and T-cells. This highlights the important roles of B-cell-derived exosomes in asthmatic inflammation as they can bypass the need for direct cell-to-cell contact [56].

Like other immune cells, T-lymphocytes can release exosomes [58–60]. Cytotoxic CD8+ T-cells release granules containing cytolysis mediators [60]. However, the bioactivity and potential immune-regulatory effect of T-cell-derived exosomes is not clear [61, 62]. Exosome released by T-cells is a selective and highly regulated process since T-cell receptor (TCR) activation, but not stimulation with mitogenic signals such as phorbol esters, greatly increases exosome production [58].

Exosomes released by activated CD4+ T-cells suppress the cytotoxic responses and antitumor immunity by CD8+ T-lymphocytes. These activated T-cells release 5–100 nm saucer-shaped exosomes that contain many proteins including lysosomal-associated membrane protein 1 (LAMP-1) and lymphocyte function associated antigen-1 (LFA-1) as well as CD4+ T-cell markers such as CD4, TCR, CD25, and Fas ligand [63]. Recent studies emphasize the importance of lipids in mediating T-cell-derived exosome production and function. These exosomes are enriched in sphingomyelin and cholesterol [64] and ceramide, tetraspanins, and myelin and lymphocyte (MAL) protein are important in T-cell exosome biogenesis [61]. MAL is a 17 KDa hydrophobic proteolipid located in the endoplasmic reticulum of T-cells and is involved in T-cell signal transduction. MAL was initially thought to be expressed only in T-cells but later was found also in myelin-forming cells and in polarized epithelial cells where it has a role in the apical transport of secretory proteins [65]. Activated CD3+ T-cells also release biologically active exosomes. These exosomes together with IL-2 triggered the proliferation of autologous resting CD3+ T-cells and induced a distinct cytokine profile [63].

In addition, several studies have shown that exosomes originating from other cell types can modulate T-cell function and subsequently affect the allergic asthmatic response [66–68]. For example, exosomes originating from B-cells [15], DCs [32], and epithelial-derived BALF exosomes [43] trigger T2 cytokine production along with increased proliferation and activation.

*Mast cells* are key immune cells in the development of allergic reactions and Th2 responses [69]. Activation of mast cells leads to the release of bioactive mediators such as histamine, prostaglandins, and LTs which subsequently trigger the allergic response. Mast cells also contribute to the secretion of proinflammatory cytokines such as TNF-*α* and IL-13 which drive the innate and adaptive immune responses in asthma [70, 71].

Mast cells constitutively release exosomes which have downstream effects on other immune cell types. For example, mast cell-derived exosomes induce DCs to acquire costimulatory MHC class II, CD80, CD86, and CD40 molecules enabling them to have an antigen presenting capacity for T-cells [72]. These exosomes can also modulate the activation of B- and T-lymphocytes and stimulate the production of cytokines such as IL-2, IL-12, and IFN-*γ* by these cells [73].

Mast cell-derived exosomes can enable target cell signaling from cell surface receptors upon contact with immune effector cells. For example, mast cell-derived exosomes trigger IgE production by B-cells in the absence of T-cells through their CD40 surface ligand [74]. Moreover, exosomes originating from bone marrow-derived mast cells (BMMCs) contain CD63 and OX40L on their surface and so can ligate with OX40 on the surface of T-cells and induce T-cell proliferation and differentiation of naïve T-cells to Th2 cells. BMMC-derived exosomes modulate the airway inflammation and remodeling responses seen in murine models of allergic asthma [75]. Mast cell-derived exosomes carry Fc*ε*RI which can bind to free IgE. This can result in decreased serum levels of IgE and limit the effects of mast cell activation. This indicates the potential of mast cell-derived exosomes as a novel anti-IgE factor in controlling the pathogenesis of severe asthma [75].

Lastly, mast cell-derived exosomes can also modulate T-cell function by donation of their contents [76] and induce the secretion of proinflammatory cytokines by human airway smooth muscle cells (ASMCs) which leads to preservation of asthmatic features [77].

*Basophiles* are a population of basophilic leukocytes and are like mast cells in that they are granular and are involved in allergic immune responses [78]. Basophiles comprise 0.5–1% of circulating white blood cells; however, upon inflammatory or chemotactic stimuli they increase in number and are recruited to the site of infection, for example. As with mast cells, basophils modulate the immune response by affecting other immune effector cells. Basophils can induce the proliferation and survival of naïve B-cells and direct their differentiation into antibody-producing cells. The crosstalk between these cells can be mediated via direct cell-to-cell contact as well as through soluble mediators and exosomes [79]. It was known for a long time that basophils release granules that resemble exosomes [80]; however there is limited evidence of exosome production by basophils [78].

*Dendritic cells* are specialized effector cells in the immune system. Acting as antigen presenting cells (APC) they process and present antigens to T-cells as well as having the capacity to phagocytose dead cells and bacteria and thereby contribute to innate immunity [81, 82]. Exosomes derived from DCs resemble their parent's morphology by possessing MHC classes I and II molecules on their surface enabling them to stimulate T-cell responses [66] or they may be captured by other APCs to induce immune responses [66]. DC-derived exosomes can present allergens and trigger the induction of Th2 responses [83]. For example, exosomes released from DCs obtained from subjects allergic to cat dander induce IL-4 responses in peripheral blood mononuclear cells (PBMCs) [38].

DC-derived exosomes contain HLA-DR, MHC, CD86, and CD54 on their surface. The presence of the costimulatory molecule CD86 indicates the potential of these exosomes to induce T-cell proliferation and differentiation whilst CD54 enables exosomes to interact with T-lymphocytes via LFA-1 [84]. These exosomes also contain enzymes that can convert LT A4 to other LTs such as LTB4 and LTC4 [84].

These exosomes also contribute to the recruitment and migration of granulocytes and leukocytes to the site of inflammation. This process is mediated by metabolites of arachidonic acid (5-keto eicosatetraenoic acid, KETE, and LTB4) that are produced following transfer of exosome-derived enzymes. These proinflammatory lipid metabolites are important in triggering asthma pathogenesis [84].

### 4.2. Exosomes from the Lung Structural Cells

Exosomes released from structural lung cells also contribute to fine-tuning of the immune response in asthma via managing intercellular communication [8]. Exosomes released by bronchial fibroblasts can be taken up by bronchial epithelial cells. Intriguingly, although the levels of transforming growth factor- (TGF-) *β*2 in exosomes derived from severe asthmatic fibroblasts were lower than that in exosomes derived from healthy subjects, fibroblast-derived exosomes from severe asthmatics induced increased proliferation of epithelial cells. The level of TGF-*β*2 in the fibroblast-derived exosomes was significantly related to the level in the cell of origin which controlled the exosome effect on bronchial epithelial cell proliferation. Thus, modulation of fibroblast TGF-*β*2 levels by overexpression or knockdown had concomitant effects on exosome levels of TGF-*β*2 and on epithelial cell proliferation [85].

The production of exosomes by lung cells and their protein content was higher in a mouse model of asthma. In this model IL-13 augmented the secretion of exosomes by lung epithelial cells and these exosomes enhanced the proliferation and differentiation of macrophages. Inhibition of exosome production by GW4869 alleviated the induction of asthmatic features in this model [44].

## 5. Exosomal miRNAs in Severe Asthma Pathogenesis

Exosomes as important mediators of cell communication can deliver miRNAs from one cell to a distinct target cell at a neighboring or distal site and subsequently affect the function of the target cell [86]. miRNAs modulate both innate and adoptive immune response with miR-21, miR-146a, and miR-155 being reported as key miRNAs in the asthmatic immune response [87]. Each miRNA can target hundreds of genes; so any changes in miRNAs level can influence many signaling pathways and have profound effects on disease pathogenesis [88].

In asthma, dysregulated miRNA expression has been observed in many cells and compartments including airway biopsies, lymphocytes, epithelial cells, and peripheral blood [89]. For example, in a murine model of asthma upregulation miR-21 was associated with altered IL-12 expression and a heightened Th2 response [90, 91]. Overexpression of miR-21 along with miR-126 was also detected in airway epithelial cells of asthmatic patients [92]. Other dysregulated miRNAs include miR-1248, miR-let7a, miR-570, miR-133a, and miR-328 which are decreased in plasma of asthmatic patient [93] whilst miR-221 was increased in ASMC from patients with severe asthma and regulated ASM proliferation and the secretion of proinflammatory mediators such as IL-6 [94].

Similarly, the exosomal miRNA content may also be altered in pathological conditions. For example, an analysis of BAL-derived exosomal miRNAs in asthma reveals the altered expression of 24 miRNAs in asthmatic patients compared to healthy subjects which are implicated in the regulation of IL-13-mediated functions [95]. In addition, CD8+ cells released exosome like vesicles that contain miR-150 and are coated with antigen specific antibody [87]. Internalization of these vesicles by the T-cells leads to antigen specific tolerance in mice [87].

Analysis of circulating exosomal miRNAs by next-generation sequencing demonstrated upregulation of miR-128, miR-140-3p, miR-196b-5p, and miR-486-5p in severe asthma patients in comparison to healthy subjects. These differentially expressed miRNAs were mostly involved in ErbB signaling pathway and focal adhesion [42]. In another study, the altered severe asthma exosomal miRNA content was associated with airway epithelial cell integrity and feature of asthma such as peripheral blood granulocyte counts [41].

## 6. The Therapeutic Potential of Exosomes in Asthma

Exosomes can regulate homeostasis and vital immune functions in the lung microenvironment. Exosomal contents have recently been suggested as potential diagnostic biomarkers in multiple diseases. In addition, as described above, exosomes can act as traps to prevent immune activation. Mast cell-derived exosomes possess FC*ε*R1 on their surface which can bind free serum IgE and limit the effects of mast cell activation [75]. Furthermore, CD8+ cells release microvesicles that contain miR-150 which can suppress allergic contact dermatitis (ACD) and induce an antigen specific tolerance in mice [87].

Mesenchymal stem cells (MSC) release exosomes with the capacity to accelerate wound healing and lung tissue regeneration and this may be of use in alleviating airway remodeling in asthma [96]. These exosomes also have antiapoptotic and anti-inflammatory properties indicating that they may be effective in other lung chronic inflammatory conditions [97]. Clinical trials using exosome-based therapy in acute respiratory distress syndrome (ARDS) are being conducted [98]. In an animal model of ARDS, exosomes derived from MSC reduce lung inflammation via induction of keratinocyte growth factor (KGF) expression in the injured alveolus and thereby improve the lung protein permeability [99].

Immunotherapy for non-small-cell lung cancer (NSCLC) using dexosomes is also undergoing clinical trials. Dexosomes are DC-derived exosomes that are loaded with tumor antigen [100]. The data to date indicate that exosome therapy is feasible and safe and may represent an alternative approach to traditional therapeutic methods in inflammatory diseases such as asthma. Further studies are required to examine the effect of exosomes on the different pathological features associated with patients with distinct phenotypes of severe asthma.

## 7. Conclusion

In recent years, exosomes have emerged as an important area in biomedical research. Exosomes play a key role in local and distant intracellular communication and have been implicated as having a crucial role in the regulation of normal cellular function and increasingly in pathological conditions. These nanovesicles are also being increasingly recognized as potentially powerful tools for the prognosis, diagnosis, monitoring, and treatment of patients in many therapeutic areas.

Within the lung microenvironment cell-to-cell communication is of upmost importance. In asthma, exosomes can regulate immune and inflammatory responses in a beneficial and detrimental manner. The severity of asthma has been linked with distinct exosomal pools and/or content which have important roles in disease at least in primary cells and in in vivo models of disease. In addition, the unique constituents of exosomes indicate their potential as biomarkers or as novel therapeutic agents. However, there are still many unsolved problems in the area including the selectively packaging of exosomal content and the mechanisms involved in the precise delivery to target cells; these need to be elucidated.

## References

[1] Fireman P. Understanding asthma pathophysiology.

[2] Subbarao P., Mandhane P. J., Sears M. R. (2009). Asthma: Epidemiology, etiology and risk factors.

[3] Lambrecht B. N., Hammad H. (2014). The immunology of asthma.

[4] Lötvall J., Akdis C. A., Bacharier L. B. (2011). Asthma endotypes: a new approach to classification of disease entities within the asthma syndrome.

[5] Lin T.-Y., Poon A. H., Hamid Q. (2013). Asthma phenotypes and endotypes.

[6] Stirling R. G., Chung K. F. (2001). Severe asthma: Definition and mechanisms.

[7] Bellanti J. A., Settipane R. A. (2015). Addressing the challenges of severe asthma.

[8] Alipoor S. D., Mortaz E., Garssen J., Movassaghi M., Mirsaeidi M., Adcock I. M. (2016). Exosomes and Exosomal miRNA in Respiratory Diseases.

[9] Alipoor S. D., Mortaz E., Tabarsi P. (2017). Bovis Bacillus Calmette-Guerin (BCG) infection induces exosomal miRNA release by human macrophages.

[10] Azmi A. S., Bao B., Sarkar F. H. (2013). Exosomes in cancer development, metastasis, and drug resistance: a comprehensive review.

[11] Dörsam B., Reiners K. S., von Strandmann E. P. (2018). Cancer-derived extracellular vesicles: Friend and foe of tumour immunosurveillance.

[12] Schorey J. S., Cheng Y., Singh P. P., Smith V. L. (2015). Exosomes and other extracellular vesicles in host-pathogen interactions.

[13] Fleming A., Sampey G., Chung M.-C. (2014). The carrying pigeons of the cell: exosomes and their role in infectious diseases caused by human pathogens.

[14] Hosseini H. M., Fooladi A. A. I., Nourani M. R., Ghanezadeh F. (2013). The Role of exosomes in infectious diseases.

[15] Sastre B., Cañas J. A., Rodrigo-Muñoz J. M., del Pozo V. (2017). Novel modulators of asthma and allergy: Exosomes and microRNAs.

[16] Tsicopoulos A., De Nadai P., Glineur C. (2013). Environmental and genetic contribution in airway epithelial barrier in asthma pathogenesis.

[17] Melén E., Pershagen G. (2012). Pathophysiology of asthma: Lessons from genetic research with particular focus on severe asthma.

[18] Busse W. W., Banks-Schlegel S., Wenzel S. E. (2000). Pathophysiology of severe asthma.

[19] Barrios R. J., Kheradmand F., Batts L., Corry D. B. (2006). Asthma: Pathology and pathophysiology.

[20] Schreck D. M. (2006). Asthma pathophysiology and evidence-based treatment of severe exacerbations.

[21] Bradding P., Walls A. F., Holgate S. T. (2006). The role of the mast cell in the pathophysiology of asthma.

[22] Gosens R., Zaagsma J., Meurs H., Halayko A. J. (2006). Muscarinic receptor signaling in the pathophysiology of asthma and COPD.

[23] Mauad T., Bel E. H., Sterk P. J. (2007). Asthma therapy and airway remodeling.

[24] Beasley R., Page C., Lichtenstein L. (2002). Airway remodelling in asthma.

[25] Raposo G., Stoorvogel W. (2013). Extracellular vesicles: exosomes, microvesicles, and friends.

[26] Bhatnagar S., Schorey J. S. (2007). Exosomes released from infected macrophages contain *Mycobacterium avium* glycopeptidolipids and are proinflammatory.

[27] Singh P. P., Smith V. L., Karakousis P. C., Schorey J. S. (2012). Exosomes isolated from mycobacteria-infected mice or cultured macrophages can recruit and activate immune cells in vitro and in vivo.

[28] Kim C.-H., Hong M.-J., Park S.-D. (2006). Enhancement of anti-tumor immunity specific to murine glioma by vaccination with tumor cell lysate-pulsed dendritic cells engineered to produce interleukin-12.

[29] Singh P. P., LeMaire C., Tan J. C., Zeng E., Schorey J. S. (2011). Exosomes released from m.tuberculosis infected cells can suppress ifn-*γ* mediated activation of naïve macrophages.

[30] Valadi H., Ekström K., Bossios A., Sjöstrand M., Lee J. J., Lötvall J. O. (2007). Exosome-mediated transfer of mRNAs and microRNAs is a novel mechanism of genetic exchange between cells.

[31] Takahashi A., Okada R., Nagao K. (2017). Exosomes maintain cellular homeostasis by excreting harmful DNA from cells.

[32] Hough K. P., Chanda D., Duncan S. R., Thannickal V. J., Deshane J. S. (2017). Exosomes in immunoregulation of chronic lung diseases.

[33] Admyre C., Telemo E., Almqvist N. (2008). Exosomes—nanovesicles with possible roles in allergic inflammation.

[34] De Toro J., Herschlik L., Waldner C., Mongini C. (2015). Emerging roles of exosomes in normal and pathological conditions: new insights for diagnosis and therapeutic applications.

[35] Fujita Y., Kosaka N., Araya J., Kuwano K., Ochiya T. (2015). Extracellular vesicles in lung microenvironment and pathogenesis.

[36] Eissa N. T. (2013). The exosome in lung diseases: message in a bottle.

[37] Fujita Y., Yoshioka Y., Ito S., Araya J., Kuwano K., Ochiya T. (2014). Intercellular communication by extracellular vesicles and their microRNAs in Asthma.

[38] Admyre C., Grunewald J., Thyberg J. (2003). Exosomes with major histocompatibility complex class II and co-stimulatory molecules are present in human BAL fluid.

[39] Mazzeo C., Cañas J. A., Zafra M. P. (2015). Exosome secretion by eosinophils: a possible role in asthma pathogenesis.

[40] Prado N., Marazuela E. G., Segura E. (2008). Exosomes from bronchoalveolar fluid of tolerized mice prevent allergic reaction.

[41] Francisco-Garcia A., Martinez-Nunez R. T., Rupani H. (2016). LSC Abstract Altered small RNA cargo in severe asthma exosomes.

[42] Suzuki M., Konno S., Makita H. (2016). LSC Abstract - Altered circulating exosomal RNA profiles detected by next-generation sequencing in patients with severe asthma.

[43] Torregrosa Paredes P., Esser J., Admyre C. (2012). Bronchoalveolar lavage fluid exosomes contribute to cytokine and leukotriene production in allergic asthma.

[44] Kulshreshtha A., Ahmad T., Agrawal A., Ghosh B. (2013). Proinflammatory role of epithelial cell–derived exosomes in allergic airway inflammation.

[45] Levänen B., Bhakta N. R., Torregrosa Paredes P. (2013). Altered microRNA profiles in bronchoalveolar lavage fluid exosomes in asthmatic patients.

[46] Barnes P. J. (2008). Immunology of asthma and chronic obstructive pulmonary disease.

[47] Barnes P. J. (2008). The cytokine network in asthma and chronic obstructive pulmonary disease.

[48] Hammad H., Lambrecht B. N. (2008). Dendritic cells and epithelial cells: linking innate and adaptive immunity in asthma.

[49] Cañas J. A., Sastre B., Mazzeo C. (2017). Exosomes from eosinophils autoregulate and promote eosinophil functions.

[50] Medoff B. D., Thomas S. Y., Luster A. D. (2008). T cell trafficking in allergic asthma: The ins and outs.

[51] Anderson G. P. (2008). Endotyping asthma: new insights into key pathogenic mechanisms in a complex, heterogeneous disease.

[52] Lindell D. M., Berlin A. A., Schaller M. A., Lukacs N. W. (2008). B cell antigen presentation promotes Th2 responses and immunopathology during chronic allergic lung disease.

[53] De Vooght V., Carlier V., Devos F. C. (2013). B-lymphocytes as key players in chemical-induced asthma.

[54] Harris D. P., Haynes L., Sayles P. C. (2000). Reciprocal regulation of polarized cytokine production by effector B and T cells.

[55] Natarajan P., Guernsey L. A., Schramm C. M. (2014). Regulatory B cells in allergic airways disease and asthma.

[56] Admyre C., Bohle B., Johansson S. M. (2007). B cell-derived exosomes can present allergen peptides and activate allergen-specific T cells to proliferate and produce TH2-like cytokines.

[57] Clayton A., Turkes A., Navabi H., Mason M. D., Tabi Z. (2005). Induction of heat shock proteins in B-cell exosomes.

[58] Blanchard N., Lankar D., Faure F. (2002). TCR activation of human T cells induces the production of exosomes bearing the TCR/CD3/*ζ* complex.

[59] Peters P. J., Geuze H. J., van der Donk H. A. (1989). Molecules relevant for T cell-target cell interaction are present in cytolytic granules of human T lymphocytes.

[60] Peters P. J., Borst J., Oorschot V. (1991). Cytotoxic T lymphocyte granules are secretory lysosomes, containing both perforin and granzymes.

[61] Ventimiglia L. N., Alonso M. A. (2016). Biogenesis and Function of T Cell-Derived Exosomes.

[62] Zhang H., Xie Y., Li W., Chibbar R., Xiong S., Xiang J. (2011). CD4 T cell-released exosomes inhibit CD8 cytotoxic T-lymphocyte responses and antitumor immunity.

[63] Wahlgren J., Karlson T. D. L., Glader P., Telemo E., Valadi H. (2012). Activated Human T Cells Secrete Exosomes That Participate in IL-2 Mediated Immune Response Signaling.

[64] Bosque A., Dietz L., Gallego-Lleyda A. (2016). Comparative proteomics of exosomes secreted by tumoral Jurkat T cells and normal human T cell blasts unravels a potential tumorigenic role for valosin-containing protein.

[65] Martin-Belmonte F., Arvan P., Alonso M. A. (2001). MAL Mediates Apical Transport of Secretory Proteins in Polarized Epithelial Madin-Darby Canine Kidney Cells.

[66] Théry C., Duban L., Segura E., Væron P., Lantz O., Amigorena S. (2002). Indirect activation of naïve CD4^+^ T cells by dendritic cell-derived exosomes.

[67] Clayton A., Al-Taei S., Webber J., Mason M. D., Tabi Z. (2011). Cancer exosomes express CD39 and CD73, which suppress T cells through adenosine production.

[68] Abusamra A. J., Zhong Z., Zheng X. (2005). Tumor exosomes expressing Fas ligand mediate CD8+ T-cell apoptosis.

[69] Reuter S., Stassen M., Taube C. (2010). Mast cells in allergic asthma and beyond.

[70] Bradding P., Brightling C. (2007). Mast cell infiltration of airway smooth muscle in asthma.

[71] Rossios C., Pavlidis S., Gibeon D. (2017). Impaired innate immune gene profiling in airway smooth muscle cells from chronic cough patients.

[72] Skokos D., Botros H. G., Demeure C. (2003). Mast cell-derived exosomes induce phenotypic and functional maturation of dendritic cells and elicit specific immune responses in vivo.

[73] Tkaczyk C., Villa I., Peronet R., David B., Chouaib S., Mécheri S. (2000). In vitro and in vivo immunostimulatory potential of bone marrow-derived mast cells on b- and T-lymphocyte activation.

[74] Gauchat J.-F., Henchoz S., Mazzei G. (1993). Induction of human IgE synthesis in B cells by mast cells and basophils.

[75] Xie G., Yang H., Peng X. (2017). Mast cell exosomes can suppress allergic reactions by binding to IgE.

[76] Li F., Wang Y., Lin L. (2016). Mast Cell-Derived Exosomes Promote Th2 Cell Differentiation via OX40L-OX40 Ligation.

[77] Xia Y. C., Harris T., Stewart A. G., MacKay G. A. (2012). Secreted factors from human mast cells trigger inflammatory cytokine production by human airway smooth muscle cells.

[78] Stone K. D., Prussin C., Metcalfe D. D. (2010). IgE, mast cells, basophils, and eosinophils.

[79] Merluzzi S., Betto E., Ceccaroni A. A., Magris R., Giunta M., Mion F. (2014). Mast cells, basophils and B cell connection network.

[80] Dvorak A. M. (2005). Degranulation and recovery from degranulation of basophils and mast cells, in Ultrastructure of Mast Cells and Basophils.

[81] Gu X., Erb U., Büchler M. W., Zöller M. (2015). Improved vaccine efficacy of tumor exosome compared to tumor lysate loaded dendritic cells in mice.

[82] Zitvogel L., Mayordomo J. I., Tjandrawan T. (1996). Therapy of murine tumors with tumor peptide-pulsed dendritic cells: Dependence on T cells, B7 costimulation, and T helper cell 1-associated cytokines.

[83] Vallhov H., Gutzeit C., Hultenby K., Valenta R., Grönlund H., Scheynius A. (2015). Dendritic cell-derived exosomes carry the major cat allergen Fel d 1 and induce an allergic immune response.

[84] Esser J., Gehrmann U., D'Alexandri F. L. (2010). Exosomes from human macrophages and dendritic cells contain enzymes for leukotriene biosynthesis and promote granulocyte migration.

[85] Haj-Salem I., Plante S., Gounni A. S., Rouabhia M., Chakir J. (2018). Fibroblast-derived exosomes promote epithelial cell proliferation through TGF-*β*2 signalling pathway in severe asthma.

[86] Théry C., Zitvogel L., Amigorena S. (2002). Exosomes: composition, biogenesis and function.

[87] Rebane A., Akdis C. A. (2014). MicroRNAs in allergy and asthma.

[88] Alipoor S. D., Adcock I. M., Garssen J. (2016). The roles of miRNAs as potential biomarkers in lung diseases.

[89] Jiang X. (2011). The emerging role of microRNAs in asthma.

[90] Lu T. X., Munitz A., Rothenberg M. E. (2009). MicroRNA-21 is up-regulated in allergic airway inflammation and regulates IL-12p35 expression.

[91] Lu T. X., Hartner J., Lim E.-J. (2011). MicroRNA-21 limits in vivo immune response-mediated activation of the IL-12/IFN-*γ* pathway, Th1 polarization, and the severity of delayed-type hypersensitivity.

[92] Wu X.-B., Wang M.-Y., Zhu H.-Y., Tang S.-Q., You Y.-D., Xie Y.-Q. (2014). Overexpression of microRNA-21 and microRNA-126 in the patients of bronchial asthma.

[93] Szymczak I., Wieczfinska J., Pawliczak R. (2016). Molecular Background of miRNA Role in Asthma and COPD: an updated insight.

[94] Perry M. M., Baker J. E., Gibeon D. S., Adcock I. M., Chung K. F. (2014). Airway smooth muscle hyperproliferation is regulated by MicroRNA-221 in severe asthma.

[95] Hu Z., Chen J., Tian T. (2008). Genetic variants of miRNA sequences and non-small cell lung cancer survival.

[96] Porro C., Lepore S., Trotta T. (2010). Isolation and characterization of microparticles in sputum from cystic fibrosis patients.

[97] Huang L., Ma W., Ma Y., Feng D., Chen H., Cai B. (2015). Exosomes in mesenchymal stem cells, a new therapeutic strategy for cardiovascular diseases?.

[98] Wilson J. G., Liu K. D., Zhuo H. (2015). Mesenchymal stem (stromal) cells for treatment of ARDS: a phase 1 clinical trial.

[99] Zhu Y. G., Feng X. M., Abbott J. (2014). Human mesenchymal stem cell microvesicles for treatment of Escherichia coli endotoxin-induced acute lung injury in mice.

[100] Morse M. A., Garst J., Osada T. (2005). A phase I study of dexosome immunotherapy in patients with advanced non-small cell lung cancer.

